# Supplementary material for: Are carbon emissions trading and green financial instruments synergistic? -Comprehensive quantitative research based on content analysis
Source: PLoS One. 2024 Mar 7;19(3):e0298601. doi: 10.1371/journal.pone.0298601 (PMC10919601; doi:10.1371/journal.pone.0298601)
Supplement: S1 Data — (ZIP) [file pone.0298601.s001.zip › Data/地级市Z_F工作报告.zip!/░▓╗╒/│n╓▌╩╨/└┤╫╘╣1⁄2╓┌║┼í╛┬φ┐╦╩2╛▌═°í┐_2010.docx]

一、2009年工作回顾

　　各位代表：

　　现在，我代表市人民政府，向大会作政府工作报告，请予审议，并请市政协委员和其他列席人员提出意见。

　　一、2009年工作回顾

　　2009年，是滁州干部群众应对危机、化危为机的一年，是滁州上下各方迎难而上、奋力爬坡的一年，是滁州经济社会加快发展、全面突破的一年，是滁州外界影响持续扩大、广获好评的一年。一年来，全市上下在省委、省政府的正确领导和市委的直接领导下，以科学发展观为指导，紧紧围绕“双超”和“追宿迁、超千亿、进前列”的目标，打好“三张牌”，建设“三个园”，锐意进取，奋力拼搏，较好地完成了市四届人大二次会议确定的各项任务。

　　（一）主要指标全面突破，在实现“双超”发展目标上取得了重要进展

　　预计全市实现生产总值595亿元，比上年增长13.5%，超年度计划2.5个百分点，超全省平均增长水平；财政收入68.3亿元，增长22.9%，超年度计划7.9个百分点，超全省平均增幅5.9个百分点；全社会固定资产投资530亿元，增长47.2%，超年度计划7.2个百分点，超全省平均增幅8个百分点；金融机构新增贷款余额106亿元，比上年增长1.7倍，是年度计划的2.4倍，增幅是全省平均水平的2倍；规模以上工业增加值195亿元，增长23.9%，超年度计划6.4个百分点，超全省平均增幅2.9个百分点；社会消费品零售总额183.5亿元，增长18.8%，超年度计划2.8个百分点，超全省平均增幅0.1个百分点。主要经济指标多年来首次全面超过全省平均增长水平，生产总值、固定资产投资、银行贷款余额的增速都进入全省前列。

　　广招商、重投资，掀起了项目建设新高潮。保增长是去年工作的主旋律，扩大投资需求是实现保增长的关键举措。市政府围绕投资谋项目、抢机遇，围绕项目广招商、寻合作，积极开展“项目建设推进年”、“招商引资攻坚年”等活动。一是百业并举谋项目。全市上下紧紧围绕国家和省产业振兴规划和我市现有产业、资源，集中时间和力量编制项目。在历年已有的3000多个项目的基础上，去年又编制了涵盖工业、农业、服务业和基础设施等各行各业项目1700多个，总投资1200亿元，有效地扩充和优化了“项目库”，同时狠抓重点项目的前期工作，保障项目拿得出、用得上。二是百计千方争项目。认真研究国家政策，充分利用人脉资源，向上争取项目资金。共获得中央和省扩大内需项目投资15.54亿元，占全省十分之一以上。新增省“861”行动计划项目182个，总投资1631亿元，规模居全省第二位。大唐联合电厂、滁河综合治理、宁滁城际轻轨等对滁州未来发展具有较大牵动力的项目前期工作取得重大进展。三是百团出击招项目。全市共组织招商分局和小分队457个，专兼职招商人员近千人，常年在外招商，全年招商引资到位资金420亿元，比上年增长35%。四是百日行动引项目。积极开展与中央企业合作，8月下旬开始大干一百天，争取中央企业来我市投资，共签约合作项目32个、协议总投资778亿元，分别居全省第二位和第五位。五是“三百”攻坚上项目。自九月份开始集中开展“百个在谈项目抓签约、百个签约项目抓开工、百个在建项目抓投产”的“三百”攻坚行动，强力推进项目的上马、开工和运营，新合作签约1000万元以上项目332个，新开工建设项目328个，新竣工投产项目300个。同时也支持、激发和推动了本土企业的投资兴业。六是百强共育大项目。全市新批和结转亿元以上项目204个，其中5亿元以上项目31个；世界500强ABB、西门子和GLG，国内的百强企业和行业的龙头企业如康佳、碧桂园、盼盼、雨润、榄菊、中材、中建材、晋煤等企业集团纷纷落户滁州或在滁扩大投资，这些知名企业集团驻滁发展，对培育“太阳级”工程、建设“月亮型”项目、实现“千亿元”产业和吸引更多投资商落户滁州，促进产业招商、以商引商，意义重大。

　　　保企业、壮支柱，促进了工业经济上台阶。保企业就是保增长成为全市上下共识。全年工业增加值突破200亿元，达到230亿元，增长20%，实现利润22.5亿元，增长17.2%。全市一、二、三产业增加值的比重达到22.7:44.7:32.6，其中二产对经济增长的贡献率达到62.5%。面对去年上半年工业经济发展的颓势，迎难而上，主动作为，多管齐下。一是帮扶解困一批。召开高规格动员大会，出台帮扶政策，派出解困工作组，密集开展银政企对接，实施“五缓四减三补贴”政策，为企业减负7664万元，全年工业企业亏损面下降了1.5个百分点。二是招商发展一批。预计全年引进工业项目1551个，总投资921亿元，500万元以上项目新投产516个。规模以上工业企业突破1000户，达到1007户，其中天长市达到268户，来安县、全椒县和市开发区都分别超过100户。三是技改壮大一批。全年技改完成投资130亿元，增长46.1%。天大石油管材二期、长丰扬子皮卡车、安兴彩纤、富达电机、明光润海高科、来安金禾精细化工、琅琊华润啤酒、凤阳华众玻璃等项目建成投产，定远盐化工、全椒海螺水泥、南谯恒昌机械等项目加快推进。全市产值超亿元企业由上年121户上升到140户，天大、博西华、全柴、滁烟4户企业超20亿元。四是规划集聚一批。先后制定了家电信息、石英硅能、盐化工三个千亿元产业规划和机械汽车、农副产品深加工等一批百亿元产业规划，吸引了新申花、杰事杰、金帅、台玻等龙头企业入驻，促进了产业集聚。支柱产业中有3个产业产值超过百亿元，其中家电信息产业130亿元（规模以上企业达131家），机械汽车产业190亿元，农副产品加工业115亿元。五是园区扩充一批。市开发区二期建设全面启动，来安汊河、天长秦栏、凤阳板桥3个工业园区被批准筹建省级工业园区，全市省级开发区和工业园区总数达到12个，开发面积达到75平方公里；琅琊工业集中区完成了规划调整并启动建设，各地通过培植经济强镇建设了一批乡镇工业集中区。

　　抓农业、强基础，保障了农村经济稳发展。农业生产能力进一步增强。大力推进“百亿粮仓”建设，加强农田水利基本建设，继续推进江淮分水岭综合治理开发，不断提高农机化水平，农业生产获得新丰收。粮食总产84.5亿斤，再创历史新高；肉类总产33.1万吨，水产品总产27万吨，分别比上年增长6.9%和7%。农业经济结构进一步优化。优质粮油生产扩大到80%，畜禽规模养殖比重提高到65%以上，养殖业在大农业中的比重达到42.5%，完成植树造林11.9万亩，苗木花卉发展到6万亩。农业产业化经营进一步推进。雨润集团定远县200万头生猪和全椒县3000万只家禽屠宰加工、正大100万头生猪产业化示范园、隆平高科100万亩超级稻示范基地和盼盼、乐天、威廉等项目相继签约或开工建设。农民专业合作社超过1000个，无公害农产品、绿色食品、有机食品发展到275个，都位居全省前列。农村发展亮点进一步增多。各地把村庄归并、土地整治和建设用地置换作为推进新农村建设的重要抓手，全年完成土地置换近3万亩，走在全省前列；中小水库除险加固数量占全省一半；解决了18.5万农村人口安全饮水问题，定远县“集中供水”模式受到水利部肯定，被中央电视台新闻联播报道，引发全国关注；17个镇被列入省扩权强镇试点，总量居全省第二；农民收入突破5000元大关，首次赶上了全国平均水平，达到5038元，增长10.9%，超出全省平均水平400元以上。劳务收入对农民增收的贡献超过50%。农业和农村经济的稳步发展巩固了县域经济基础。全年县域财政收入达43亿元，增幅高于全市平均水平2个百分点，占全市财政收入比重63%，提高1个百分点；12个乡镇财政收入超过5000万元，其中天长市铜城镇突破3亿元。天长市成为全省唯一的连续十年十强县。

　　活流通、拓服务，形成了三产发展好氛围。一手抓传统商贸流通的提升。深入开展“万村千乡”农家店建设和“家电下乡”、地产工业品对接等活动，实现家电下乡销售额2.7亿元。一手抓旅游、物流、金融、房地产等现代服务业的拓展。加快发展旅游业。组建了旅游经济和琅琊山旅游区建设指挥部，成立琅琊山风景区管委会，将琅琊山旅游区范围从115平方公里扩大到240平方公里；九天峰等旅游度假项目建设继续推进，建成白鹭岛温泉公园和舜耕生态农业园一期工程，凤阳韭山森林公园获批国家地质公园，小岗村获批国家4A级景区，金地大酒店晋升为五星级酒店；全年接待游客513万人次，实现旅游收入32.4亿元。积极推进物流业。皖东国际车城、金太阳国际装饰城、宇航物流园等建设加快推进，华东农贸食品城、瑶海农机大市场、五洲国际商城、财富广场、意大利名品商贸中心等相继开工建设。大力拓展金融业。全市成立14家中小企业贷款担保公司，担保额达12亿元；开业和批准筹建、申报筹建的小额贷款公司12家，徽商银行天长支行开业，滁州和明光农村合作银行通过初审上报待批筹建。金融保险机构和保险业务持续扩大。稳步发展房地产业。举办首届房地产业高峰论坛，出台相关支持政策。全市房地产业累计完成投资66亿元，同比增长35%；新开工面积250万平方米，同比增长42%；销售面积160万平方米，同比增长75%。

　　（二）城乡建设强力推进，在统筹城乡共同发展上取得了重大进展

　　大滁城建设如火如荼。2009年，大滁城“131”组团完成投资61亿元，超过“九五”“十五”累计投资的总和。规划修编成果丰硕。聘请了院士级著名专家担任顾问，编制了清流河景观带、丰乐亭景区、龙蟠河公园、城南新区核心区、高铁站前区、城东工业园、城北新区、南谯新区、“滁来全”空间管制等9项区域性规划和城市公交、供排水、教育、卫生等19项专项规划。城市路网进一步完善。新建续建城市道路158公里，城区路网覆盖面积达到60平方公里；高标准完成了滁全路景观大道改造和滁宁快速通道滁州段建设，为“131”组团发展和与南京同城化发展创造了条件。城市功能进一步完善。滁州中学、实验小学新校区等项目加快建设，武警支队新营区、龙蟠河主题公园、菱溪湖公园、“一校两团”（市艺术学校、歌舞团、戏剧团）等工程相继开工，西撇洪沟具备了行洪能力，明光路跨线桥建设启动实施，新增城市燃气用户1.1万户，新铺供水管道50公里，新增公交车50辆。房屋拆迁、安置房建设和商品房开发均超历史。广大市民顾全大局，以实际行动支持大滁城建设，拆迁面积和安置房建设面积双双突破百万平方米。国有土地出让7000亩，土地出让金12.9亿元，是前三年的总和。房地产投资25亿元，新开工面积120万平方米。片区开发改造步伐加快。城南新区政务中心等公共设施和居民小区建设加快推进；老城区高标准实施了小铁路拆迁改造和琅琊古道改造，高杆灯、原滁州剧院、原二纺机、西水银庄等改造建设稳步实施，南湖改造二期拆迁建设全面启动。深入开展“五城联创”活动，新增城市绿化120万平方米，超过建国以来绿化工程面积的总和。运用现代技术对琅琊大道、南湖公园等实施了亮化，加强城市管理，市容市貌不断改善。

　　县乡村建设高潮迭起。以丰乐杯城镇建设竞赛、培植经济强镇、新农村建设为抓手，加快全市城镇化步伐。大滁城三个副中心全椒县城、来安县城、南谯乌衣新区全面融入对接大滁城，路网框架迅速拉开，重点项目启动实施。新规划的琅琊新区建设前期工作强力展开。其它县市也积极呼应、加快建设。天长市总投资15亿元的34个城建重点项目相继开工，明光市新明城十大重点工程加快建设，定远县城东新区现代化气息初步显现，凤阳县新城区建设进展良好。全市所有乡镇总体规划全部完成，村庄规划完成了62%，通过村庄整治建设新居民点800多个，新建沼气池5556口，新农村建设取得积极进展，城乡面貌有了新的改观。

　　水电路建设深入推进。完成水利建设投入7.3亿元，列入2008年国家、省计划的72座小型水库和10座中型水库除险加固任务全面完成，列入2009年国家、省计划的20座中型水库、70座小型水库全面开工建设，滁河堤防应急加固、沿淮洼地治理等工程全面完成。大力推进交通对接工程和村村通工程，完成交通固定资产投资11.2亿元，在建项目总数、建设里程和投资规模均居全省第一。104国道滁汊段、205省道天冶段、合蚌路定凤段、滁定路改造基本完成；明徐高速开工建设，滁马高速和蚌淮高速凤阳段前期工作进展顺利；修建“村村通”水泥路1381公里，提前一年完成“十一五”规划。电力建设完成投资5.63亿元，完成6个110千伏以上电网建设项目，开工建设110千伏以上电力项目2个。3G通信工程全面启动。

　　（三）改革开放继续深化，在创新工作体制机制上取得了积极进展

　　各项改革深入推进。市交通、建设、二轻、商业、农机等系统企业改制加速推进，成功引进省交通集团对市汽运总公司等3家运输企业进行了重组，市供销、物资等系统和汇龙电子公司、皖东水泥厂、市房地产开发公司等11家市直企业改制基本完成。各县市区企业改制力度进一步加大。积极推进行政体制改革。新一轮市政府机构改革全面启动，内部“三定”正在推进，县级政府机构改革方案已批复各县市区。完成了市区财政管理体制和城市管理体制调整。开展了市区教育管理体制改革。依法完善了食品药品安全管理体制。土地流转等农村改革进一步深化，集体林权制度改革基本完成，惠民直达工程试点深入推进。开展了司法监狱体制改革，制定了文化体制改革方案，天长市、琅琊区基层医药卫生体制省级改革试点工作进展顺利。

　　对外开放加快步伐。坚持东向发展，从东引力，与东接轨。紧紧抓住建设皖江城市带产业转移示范区机遇，高水平编制规划，努力把我市打造成为东部产业转移的“第一站”和“首选地”。市开发区先后与南京、无锡高新技术开发区和上海莘庄、漕河泾工业园区签订了合作协议，南谯区、琅琊区分别与上海浦东新区川沙功能区和宝山区大场镇签订了园区合作协议。加强对外经贸交流与合作，积极改善外贸环境。滁州海关和商检局正式获批筹建，天长市商检办事处成立。海关滁州工作组积极创新“属地申报、口岸放行”等模式，为企业通关提供便利。在全国、全省进出口大幅度下降的情况下，博西华、天大等骨干企业进出口稳中有升，稳定了全市外贸形势。年末全市外资企业189家，新增37家。预计全市进出口额完成6.88亿美元，虽然比上年下降2%，但增幅仍高于全省平均水平23个百分点，出口额进入全省前三名。

　　（四）社会事业明显加强，在经济社会协调发展上取得了显著进展

　　坚持以民生工程为抓手推动社会事业发展。省、市实施的32项民生工程投入资金26亿元，其中地方配套5.1亿元全部足额到位。优先发展教育。围绕城市建设和乡镇村撤并，调整优化教育布局，促进城乡教育尤其是义务教育均衡发展。加大对教育的投入。完善城乡义务教育经费保障机制，城乡义务教育学校教师绩效工资和学生学杂费免除、免费提供农村中小学生教科书、向农村贫困寄宿生和高校及中职学校贫困生提供资助等政策全部落实到位。招聘中小学教师820名，重点向农村倾斜。全面完成中小学校舍抗震安全排查和鉴定工作。滁州科教园区规划建设全面启动，滁州职业技术学院新校区二期、滁州学院南校区二期、一职高和县市区职业高中扩建工程加快建设。积极推动科技进步。市高新技术创业中心二期竣工，组建了家电产业公共信息平台和重点实验室，与上海交大、合肥工大等签署了产学研合作协议，全市新认定高新技术企业11家，先后获批省级、国家级现代家电高新技术产业基地。卫生事业稳步发展。全力做好甲型H1N1流感和手足口病等防控和救治工作。争取并开工建设扩大内需卫生项目78个，已竣工72个。加强医疗卫生机构标准化建设。完成27个社区卫生服务机构、28所乡镇卫生院和205个村卫生室建设任务。免费提供婚前检查12032对。开工建设两个县级妇幼保健机构。市一院、市中西医结合医院、定远县总医院、明光市中医院、天长市人民医院门诊楼等重点工程建设进展顺利。新招录卫技人员570人，主要充实农村基层。计划生育工作面对困难求突破，进一步拉升标杆、主攻后进、细化任务、狠抓督查，人口自然增长率控制在4.02％。，整体形势明显好转。发展文化广电事业。隆重举行新中国成立六十周年庆祝活动。第二届中国农民歌会和第八届安徽花鼓灯会在我市成功举办，广获好评，进一步提高了滁州的知名度。建设乡镇综合文化站20个、农家书屋303家。完成531个通电自然村广播电视开通任务。竞技体育和群众性文化体育活动双向推进。完成了67个村的农民健身工程建设。重视发展环保事业。突出抓好节能减排和污染防治，节能减排指标超额完成省下达任务。市和6个县市污水处理厂全部建成投入使用。进一步整治滁城浴室燃煤锅炉污染。军分区迁建工程进展顺利。驻军服务、国防动员、人民防空、预备役建设、双拥优抚工作得到加强。统计、气象、防震、档案、保密、地方志、妇女儿童和老龄工作投入加大、事业发展。民族、宗教和外事、侨务、工商联工作都取得了新的成绩。

　　坚持以就业和社会保障为抓手促进社会和谐。落实积极就业政策，多层次安排就业援助，建成15个农民工创业园，带动8500人就业；完成新型农民培训5.1万人次、农民工技能培训4.2万人次；发放创业小额贷款8476万元，向18户承诺不裁员企业发放补助资金850万元；全市新增就业人数3万人，城镇登记失业率保持在4.2%以内。城镇居民人均可支配收入达13296元，增长8.5%。积极扩大城乡基本保障的覆盖面。城镇职工基本养老保险和失业保险的参保人数分别扩大到22.4万人和20.3万人，城市低保和农村低保人数分别扩大到7.5万人和14.3万人；城镇居民医疗保险和新型农村合作医疗实现全覆盖，保障水平尤其是大病救助水平明显提高，城市廉租住房保障范围扩大到人均住房不足15平方米和人均月收入不足320元的家庭，开工建设廉租房30万平方米，发放廉租补贴1723万元，农村因灾倒房重建工作基本完成。积极开展社会救助，全市发放春荒救助金1000多万元；农村五保人数扩大到2.8万人，新改建敬老院38所，集中供养率提高6个百分点；向7376名农民发放了计划生育奖扶资金；15.3万大中型水库移民享受到后期扶持直补；城镇未参保集体企业退休人员全部被纳入基本生活补助范围；向3.5万名城乡重度残疾人提供生活救助2286万元；为1170名贫困白内障患者免费实施了复明手术。政策性农业保险参保面积562万亩，参保牲畜18万头。全方位、立体化的社会保障和社会救助机制正在形成。

　　坚持以服务和管理为抓手维护社会稳定。自下而上立体排查信访隐患，深入开展大接访活动，突出抓好问题解决和矛盾化解，畅通信访渠道，全市信访总量稳中有降。突出60周年国庆等重要节点抓好安保工作。保持对违法犯罪的严打攻势，集中开展禁赌、打击传销、“扫黄打非”、打击涉枪涉爆等专项行动，强化治安巡逻和视频监控建设，积极推进社会治安综合治理，“平安滁州”建设得到加强。深入开展非煤矿山、烟花爆竹和建设、交通等领域安全生产隐患排查，层层建立挂牌督办制度，妥善处置云南水富县农民工矽肺病、凤阳6•21爆炸和南洛高速滁州段10•7特大交通事故等，强化了安全生产监管。建立健全突发事件应急预案、处置体系和管理机制，有效地维护了社会大局的稳定。

　　（五）自身建设得到加强，在提高行政服务能力上取得了有效进展

　　根据市委的统一部署，政府系统深入开展了学习实践科学发展观活动，进一步增强了贯彻科学发展观的自觉性和坚定性。自觉接受和坚决服从市委的统一领导，有力促进了滁州的政治生态建设。依法行政深入推进。坚持依法接受市人大及其常委会的法律监督、工作监督，主动接受市政协的民主监督，认真听取各民主党派、工商联、无党派人士和人民团体的意见，高度重视吸纳社情民意，及时办理人大工委和政协专委的专题调研意见。市政府全年共办理人大议案2件、人大代表建议69件、人大常委会审议意见12件、政协提案325件、政协建议案5件，办结率均为100%，并努力提高办理质量；加强“五五”普法，增强法治意识；加强行政执法，突出抓好行政处罚自由裁量权的规范运用。效能建设深入开展。全面实施了重点工作推进、重大项目联系、目标绩效考评、帮办服务体系等12项体制机制创新，企业登记注册、基建联合审批、“3+1”联网服务、信息公开发布四大平台全面建成运行；取消市本级行政审批项目60项，合并20项，暂缓审批4项；对省下放的211个行政审批项目，取消36项，进一步下放29项，合并18项，暂缓审批11项；整合党委、政府和纪委督办机构，集中督办力量，创新督办机制，强力推进工作落实；加强“12345”市长热线工作，畅通政风行风热线，开辟《效能观察》栏目，增强效能舆论监督；开展基层站所评优评差活动，拓展社会监督渠道。党风廉政建设深入落实。认真落实党风廉政责任制，深入开展工程建设领域突出问题重点治理等活动，进一步扩大政府招标采购统一管理范围，加强政务公开，强化行政问责，开展监察和审计监督，查处了一批违法违纪案件。

　　2009年，我们共同经历了很多困难，我们携手战胜了国际金融危机带来的挑战，保持了经济快速发展，实现了社会和谐稳定，成绩来之不易。这是全市上下在科学发展观的正确指引和省委、省政府与市委的坚强领导下共同努力的结果，在此，我代表市人民政府，向市委、市人大、市政协、市纪委、市军分区等各大班子及其各位领导，向全市450万人民，向人大代表、政协委员和党代会代表，向离退休老同志，向各民主党派、工商联、无党派人士和各人民团体，向驻滁解放军指战员、武警消防官兵，向国家和省驻滁单位，向广大来滁投资客商和建设施工人员，向长期以来所有参与、支持和关心滁州发展的海内外朋友，表示衷心的感谢和崇高的敬意！

　　在肯定成绩的同时，我们也清醒地看到，经济社会发展中还存在不少值得重视的问题。从外部看，国际金融危机的影响还没有完全过去，宏观经济回升的基础还不巩固，将继续给我市部分行业、部分企业带来困难；随着经济逐步回暖，区域经济竞争将更加激烈。从自身看，一是经济总量不够大，结构不够优，工业缺乏大企业、大项目、高新技术项目支撑，竞争力不强，现代农业发育不足，新兴服务业发展不快，对外开放度不高，县市区之间和行业之间、园区之间发展不平衡，发展质量还有待尽快提高；二是城镇化建设虽然取得了较大的成绩，但总体水平还不高，中心城市和县城功能不够完善，带动力不够强，农村小城镇发展比较慢；三是财政实力还比较弱，保障能力不强，社会事业欠账比较多，社会保障水平不高，干部工资标准和群众收入水平还比较低，部分群众生活比较困难；四是政府自身建设还存在薄弱环节，政府机关在服务意识、工作作风上还存在形式主义、官僚主义现象，政府干部的市场经济意识、创新工作能力、应对复杂局面经验等都还存在不足，廉政建设方面也还有需要重视和改进的地方。这些困难和问题我们将认真对待，积极采取措施逐步加以解决。

　　二、2010年工作安排

　　2010年是全面完成“十一五”规划、为“十二五”打好基础的的最后一年，是实现“双超”和“大滁城”建设初见成效的关键一年，也是滁州乘势而上、力争有更大作为的重要一年。宏观形势表明，国际金融危机对实体经济的冲击在减弱，世界经济趋于回暖，国内经济持续回升，国家将继续实施积极的财政政策和适度宽松的货币政策，加快发展方式转变，加大结构调整力度，东部地区产业转移的趋势将得到延续和加快。从我市的情况看，通过近年来纵深推进工业强市、城镇化和东向发展三大战略，大力招商引资、启动民资和向上争取项目，经济连续实现两位数增长，发展的思路更加明确，发展的力量更加集聚，发展的氛围更加浓烈，已进入厚积薄发、加速崛起的新阶段，为下一步持续发展和跨越式发展奠定了良好的基础。尽管在发展中还存在这样那样的困难和问题，但我们滁州人民完全有信心、有能力战胜困难和挑战，实现经济社会发展的新突破。综合需要和可能等多方面因素，2010年全市经济社会发展的预期目标是：生产总值增长13.5%以上、争取15%，财政收入增长18%以上、争取20%，全社会固定资产投资增长30%以上、争取40%，社会消费品零售总额增长19%，外贸进出口总额增长12%以上，城镇居民人均可支配收入和农民人均纯收入分别增长12%和10%，城镇登记失业率控制在4.4%以内，人口自然增长率控制在6.5％。以内，单位生产总值能耗降低4.3%，主要污染物排放量减少4%。

　　为实现以上目标任务，2010年全市政府工作总体要求是：全面贯彻党的十七大和十七届三中、四中全会精神，以邓小平理论和“三个代表”重要思想为指导，深入贯彻科学发展观，认真落实省委、省政府的决策部署，在市委的坚强领导下，坚定不移地推进改革开放，纵深推进工业强市、城镇化和东向发展三大战略，着力打好区位、环境、资源三张牌，加大调结构、促转变力度，推进工业、城镇、农业、现代服务业“四轮”联动发展，打造承接东部产业转移的新型产业园、与南京同城发展的城市新家园、吸引东部的魅力后花园，狠抓民生工程实施，保持和拓展经济社会又好又快发展的良好势头，全力向实现“双超”和“追宿迁、超千亿、进前列”的目标迈进。

　　（一）突出新型产业园，在增强驱动力上取得新突破

　　工业化是不可逾越的历史发展阶段。我市具有发展现代大工业必需的区位、交通、资源等有利条件和国家批准建设皖江城市带承接产业转移示范区良好机遇。当前和今后一段时期，必须把建设新型产业园作为首要任务，把加快工业发展作为第一驱动力。

　　抓投入扩总量。总量不大是我市当前工业面临的最突出问题，要狠抓工业投入，千方百计做大工业总量，为结构调整创造空间。今年规模以上工业企业要达到1200家以上，新增200家，规模以上工业增加值超过240亿元。一是要加快推进在建项目。加强调度和帮办服务，促进盼盼食品、定远盐化工、全椒海螺水泥、明光榄菊日化、凤阳台玻等重大项目加快建设。二是要加快推进签约项目开工。月月抓一批项目集中开工。今年至少要开工建设1000个工业项目，确保500家以上企业投产形成现实生产力。三是要加快推进产业集聚。现代经济发展的历程告诉我们，区域经济实力取决于产业发展水平，核心竞争力取决于能否形成产业集聚效应。围绕支柱产业和重点龙头企业，从产业链条入手，重点完善家电信息、盐化工、石英（玻璃）三个千亿元产业规划，实施大企业上台阶、中企业上水平、小企业上规模的“三上”工程，扶持天大、全柴、博西华等成为百亿元企业，滁州烟厂、金禾实业等成为50亿元企业，使全市亿元企业达到160家。同时，围绕我市的资源优势、国家的政策走向和市场的发展趋势，“无中生有”，规划和培育新兴产业和企业集群，培育经济增长的“爆发点”。

　　抓技改扩体量。技术改造是推动现有产业升级的重要手段，是企业滚动发展的重要途径。要制定政策措施，通过规划引领、配套带动、贷款贴息、专项资金扶持等手段，鼓励企业大力开展技术改造，做大单个企业体量，打造一批小型“巨人企业”。今年重点实施好300个以上技术改造项目、10个国家和省级科技创新项目，完成技改投资150亿元以上，推动长丰扬子新上冲压生产线，推动康佳电子电器扩大产能，加快天大钢管三期建设，确保天康新能源、全椒天和机械、凤阳珍珠水泥、来安金禾化工、明光酒业、定远泉盛化工、南谯恒昌机械和博康注塑、琅琊霞客色织和爱瑞特成套装备、博西华MBF冰箱第二条线和滁烟综合技改等项目竣工投产。加强科技创新载体建设，大力支持企业建设研发中心，发展高新技术产品。加强产学研合作，加快上海交大滁州科技园等项目建设。推进企业信息化管理，围绕支柱产业和重点产业建设一批技术创新公共服务平台，加快推进德力玻璃、金禾化工、鲲鹏模具、新江玻璃、扬子木业等企业上市工作，争取实现新突破。

　　抓招商扩增量。招商引资是培育新兴产业、调整优化产业结构、增强长远竞争力的主要途径，是现有产业提升质量、扩张增量最直接、最有效的手段。今年要大力实施招商引资重大项目突破年行动，突出招大引强，继续推动与央企合作和对接，努力实现全市招商引资560亿元以上，其中新引进亿元以上项目100个以上，5亿元以上项目20个以上，实现50亿元以上项目新突破。一要在主导产业招商上寻求突破。围绕家电信息、机械汽车、纺织服装、化工建材、非金属矿深加工、农副产品深加工、森工木业等七大主导产业，招引行业内高端企业，主攻世界500强、中央企业和行业内领军企业，促进主导产业集群发展和改造升级。家电信息业，争取引进东菱集团、信利集团、格兰仕、澳柯玛等亿元以上项目20个；机械汽车业，推进长丰扬子汽车、全柴集团分别与广汽集团、恒天集团的合作或重组，积极做大做强，并引进配套零部件企业20家以上；硅能材料产业，引进大型精深加工企业20家以上，向新能源、新材料产业升级；同时，积极引进海天、利郎、七匹狼、杉杉等知名纺织服装企业，蜡笔小新、银鹭、雅客等著名食品加工企业，双虎家私、德尔地板等著名森工企业。二要在新兴产业招商上寻求突破。认真研究低碳经济带来的市场和政策机遇，加快发展绿色照明、风能发电、太阳能组件和新型节能建材等产业，依托机械制造配套优势加快发展装备制造业，同时，积极引进生物医药、节能环保、软件信息等新产业。三要在招商的组织领导上寻求突破。继续坚持全员招商、一岗双责，开展多种形式的招商攻坚行动。重点项目和线索由领导带队，相关部门跟进，组成招商团队，进行面对面、点对点对接。四要在创新招商方式方法上寻求突破。强化以商招商、推动商会招商、发动协会招商、开展联动招商，寻求联办“飞地”、合办“园区”，建立市内开发区之间“互招共享”的招商机制，防止招商线索的流失和内部之间相互诋毁、设防等不良现象。五要在推进项目落地上寻求突破。强化帮办服务，形成领导联系、部门帮办、重大问题会办的服务机制。在抓好工业招商主战场的同时，积极开展一产、三产和基础设施建设招商，吸引外来投资，加快建设步伐。

　　抓园区促集聚。按照建设新型产业园的要求，着力打造承接产业转移的五级平台。一是积极筹建150平方公里的滁州工业新区，加快编制总体规划和控制性详规，边筹建边招商。二是全力推进市经济技术开发区建设，着力提高市区经济的首位度。重点推进城北新区15平方公里开发，打造绿色照明、绿色食品、汽车配件、软件信息等专业园区，引导产业集聚，努力实现“三年翻两番”，尽快进入全省先进开发区行列，创建国家级经济技术开发区。三是全力推进县属11个省级工业园区建设，提高建设水平，增强承载能力，形成产业特色。争取今年全市省级工业园区开发规模增加25平方公里，总体超过100平方公里。四是全力推进乡镇工业集中区建设，重点抓好扩权强镇试点镇发展，强化加工配套能力，支持天长杨村、全椒十字等有条件的乡镇工业集中区申报省级工业园区。五是全力推进合作共建园区，建设“园中园”、专业园，在建设“飞地”经济园上实现新突破，打造承接产业转移的先行区。着力解决限制工业发展的瓶颈。继续抢抓省土地置换的政策机遇，集中人力、物力和财力，全力开展土地置换攻坚行动，确保今年完成土地置换4万亩以上。突出解决好堆场、货仓、码头等基础设施方面的薄弱环节，加快组建家电信息、机械汽车、绿色照明等产品检验中心，强化工业发展软硬件支撑。

　　抓服务促发展。发展经济，必须把政府有形的手和市场无形的手有效结合起来，增强政府的调控和服务功能。要强化对重点区域、重点领域、重点行业的监测调度，及时发现和解决问题。建立帮扶企业解困的长效机制，狠抓各项优惠政策的贯彻落实。认真抓好送农机下乡、送家电下乡等工作，积极开展地产品对接活动，帮助企业拓展销售渠道。进一步清理整顿行政事业性收费，规范中介服务收费，帮助企业减轻负担。积极搭建企业融资服务平台，开展银企对接活动，缓解企业尤其是中小企业融资难问题。结合实施干部活力工程，建立“千名干部帮千企”制度和重点企业领导帮办制度，帮助企业解决实际困难。

　　（二）突出城市新家园，在增强承载力上取得新突破

　　城镇化是社会发展的必然趋势，是助推工业化的强大动力。要抓住国家加快小城镇建设的机遇，大力实施同城化战略，内抓“131”组团之间的城镇化、外促与南京大都市的同城化、下促各县市区内部的城镇化，做好同城、山水、绿化“三篇文章”，建设宜居、宜业、宜游的城市新家园。

　　大滁城建设要初见成效。区域经济竞争直接表现为中心城市的竞争，要舞好大滁城建设这个龙头，带动全市城镇化加快推进。今年大滁城建设“131”组团争取完成投资150亿元；中心城市力争完成“五个一百”：即拆迁100万平方米、新建安置房100万平方米、新建道路100公里、开工项目100个、完成投资100亿元。倾力打造政务新区，在形成规模、聚集人气上初见成效。完成政务新区8.3平方公里范围内干支路建设，中都大道要延伸到高铁站前区；开工建设博物馆、科技馆、图书馆、规划展示馆，加快龙蟠大道高层办公建筑建设，使政务中心初具规模；加快龙蟠小区、滁州中学等6所学校、武警新营区等重点工程建设，开工建设体育中心等公共设施，加快提升新区形象。积极推进老城区成片改造，在完善功能、改变面貌上初见成效。按照成熟一片、改造一片的原则，重点改造南湖以南、南谯路以西、清流路以北区域，加快推进老水泥厂、高杆灯、凤凰湖周边等地块改造开发；以南北支干线建设和打通断头路为重点，进一步完善老城路网，加快建设明光路跨线桥、西涧路、紫薇南路贯通工程及丰乐路、凤凰西路、天长西路等延伸工程；充分挖掘老城的历史文化积淀，积极修编“两湖一河”老城区规划。加强老城区背街小巷改造和环卫、停车场建设。加快创建园林城市，在美化环境、提升品位上初见成效。按照创建园林城市的标准，合理布局城市公园、绿地及广场。加快龙蟠河主题公园、菱溪湖公园建设和南湖公园二期改造，开工建设市民广场、清流河综合治理、丰乐亭公园、会峰公园、植物园等工程，继续加大道路、街头巷尾、城市出入口等节点绿化。加快推进城市亮化工程。进一步完善城市规划，在科学布局、彰显功能上初见成效。按照宜居宜业宜游要求，进一步提高规划水平，强化功能分区。在加快推进城南新区建设和老城改造的同时，加快城北新区建设，启动高铁站前区、物流园区、科教园区、琅琊山旅游区建设，争取每个功能区建成1-2个影响大的项目。加强主副中心衔接，在整体推进、组团发展上初见成效。统筹协调主城区与全椒、来安两个县城和南谯政务新区的规划建设，加强道路、管网等基础设施对接，适时启动建设滁来全第二通道，积极推进大滁城区域公交一体化，强化一体化发展之势。同时，坚持一手抓建设，一手抓管理，深入开展“五城联创”和城市综合执法活动，完善城市管理体制，提高城市管理水平。

　　小城镇建设要再掀高潮。小城镇是城镇化体系的重要组成部分。要完善丰乐杯竞赛活动，形成小城镇建设与大滁城建设呼应之势。把县城建设作为小城镇建设的重中之重，尽快扩大规模、完善功能。连接天长新旧城区的荭草湖公园和两河景观带开发改造要基本完成，明光新明城攻坚要出形象，定远县城要加速向现代中等城市迈进，全椒县城要围绕铁路两站周边开发求突破，来安来城新城和汊河新城建设要联动推进，凤阳新城建设要彰显明文化特色并初具规模，南谯新区要完成投资30亿元。要同步推进老城区改造，完善商贸和社会服务功能。把集镇建设作为小城镇建设的重要支点，进一步完善集镇总体规划和详细规划，推进各项基础设施建设。抓好扩权强镇试点，创新集镇建设、融资与管理机制。搞好土地流转、置换，加快新农村居民点建设，促进人口向城镇集中。因地制宜发展小城镇特色产业，壮大小城镇经济实力。推进户籍制度改革，给进城入户农民平等的市民待遇。

　　城乡基础设施建设要加快推进。交通等基础设施是连接城乡的重要纽带。要按照服务大滁城、拓展大通道的要求，加快实施交通对接工程，进一步完善骨干交通网络。今年全市计划完成交通建设投资13.5亿元，加快徐明高速公路建设，开工建设滁马和蚌淮高速凤阳延伸段、312省道天长段、309省道明光至盱眙段、滁州城南客运总站，全面完成滁定路恢复改造任务，争取滁宁快速通道全线贯通，加强乡村循环道路建设，积极推进滁淮、滁扬高速公路和大西环等前期工作。要加强水运能力建设，重建汊河闸，加快建设滁州澳中、天长天海、凤阳鸿运和龙阳等码头。继续开展电力设施建设改造，完成投资10亿元以上，建设220千伏万桥开关站扩建工程、220千伏天河输变电工程、6个110千伏变电站，开工建设京沪高速铁路桑涧、腰铺配套工程。继续加强通信、邮政、广电等基础设施建设。加强城市自来水厂、污水处理厂、垃圾处理场等配套建设，积极推广定远县乡村集中供水经验。

　　（三）突出农业产业化，在增强保障力上取得新突破

　　农业在我市经济尤其是县域经济中举足轻重。要以农民增收为核心任务，以农业产业化经营为根本途径，着力提升农产品加工增值水平，着力提高龙头企业带动生产基地建设的能力，加快实现由农业大市向农业强市的跨越。

　　以“百亿粮仓”工程为牵引，实现农业生产条件新改善。全面完成25座中型水库、52座小型水库除险加固任务，开工建设滁河综合治理、淮河入江水道高邮湖大堤加固等工程，继续抓好驷马山、女山湖灌区续建配套与节水改造，继续推进江淮分水岭综合治理开发，积极推进山许、崔家湾、江巷水库前期工作。围绕“百亿粮仓”和百万亩超级稻原粮生产基地建设，谋划建设一批带动性强的大型农业项目。

　　以农业科技进步为支撑，促进农业综合生产能力新提升。以争创国家级和省级现代农业示范园为目标，着力开展水稻产业提升、小麦高产攻关、油菜丰产创建、玉米振兴计划和粮食丰产创建等五大科技行动，推广测土配方、良种培育、节水灌溉、储运保鲜、秸秆综合利用等农业新技术集成应用，发展设施农业和精准农业，引导农民开展农业标准化生产。提高大型农机具装备水平。做好动植物疫病防控工作。

　　以创新农业业态为方向，迈出农业结构调整新步伐。实施畜牧强农工程和渔业富民工程，发展畜禽规模养殖和渔业特色精养，提高养殖业贡献率。发展滁菊、茶叶、甜叶菊、蔬菜等，打造一批特色农业品牌。

　　启动百万亩造林行动，发展苗木花卉生产，建设十万亩以上苗木花卉基地，打造百亿林产工业。积极发展农业旅游和森林旅游，加快发展农业生产服务业，推动一产向二三产深度开发。

　　以培育龙头企业为重点，推进农业产业化经营新跨越。一手抓农产品转化，积极引进大型农副产品精深加工企业，支持雨润、正大、盼盼、GLG、永言、华龙等龙头企业发展；一手抓农产品流通，加快培育农产品流通企业，鼓励大型超市开展“农超对接”，因地制宜发展县乡农产品批发市场。今年要确保新增国家级龙头企业2至3家，省级10至15家。加强农民专业合作社规范化建设，做大做强一批农民专业合作社。

　　以落实惠农富民政策为基础，推动农村生产关系新调整。在稳定完善农村土地承包关系基础上，健全农村土地合理流转机制，鼓励农村土地向种田能手集中，争取今年新增土地流转10万亩；加强新型农民培训和农村劳动力职业技能培训，促进农村劳动力转移；积极发展农村小额信贷，引导农民规范发展资金互助，改善农村金融服务；稳步扩大农业政策性保险覆盖面；加强农民负担监管工作；在土地置换中注意完善置换政策，保护农民利益。

　　（四）突出新兴服务业，在增强支撑力上取得新突破

　　服务业的发展是衡量现代社会经济发达程度的重要标志。要一手提升传统服务业，一手发展现代服务业；一手加强生产性服务业，一手拓展生活性服务业；一手繁荣城市服务业，一手重视农村服务业，充分发挥第三产业的活化和带动作用，强化千亿滁州的服务支撑。

　　彰显文化旅游业的魅力。我市文化底蕴深厚，山水田林资源丰富，要以打造魅力后花园为抓手，以推进文化体制改革为动力，深化文化与旅游的结合。开展“万千百十”工程建设，加快旅游景区、接待设施和旅游产品开发，通过开发万顷山场、千湖水库、百个景区，建设十大旅游项目，培育十大旅游企业，做足做好山水文章，重点组织好琅琊山、九天峰、龙通、跃龙湖、神山寺、莲花塘、栖凤湖、龙尾山等休闲度假基地建设，新建一批中高档旅游星级酒店，培育一批精品旅游产品，推动旅游经济新突破。发挥好琅琊山在全市旅游业中的龙头作用，按照抓琅琊山、带旅游业、建后花园的思路，高水准、高品位规划好大琅琊山旅游区，引山入城，引水入城，科学定位，特色开发，错位发展。今年琅琊山开发要确保3至5个较大项目落地开工，尽快形成集聚效应。要积极推进文化管理体制改革，整合文化资源，发展文化产业和文化企业，着力打造农歌会等文化品牌。加强旅游产品开发和旅游市场营销，把旅游观光与休闲度假、购物娱乐、文艺演出、商务活动等结合起来，延伸旅游产业链，发展文化新业态。

　　挖掘现代物流业的潜力。着力打造大市场、大物流，调整优化全市商贸物流布局，建设以滁城为中心、六个县城为分中心的长江北岸区域性物流中心。滁城重点建设两大核心商圈、两大特色商业街区、四大物流园区、十大市场集群、八片区32个农贸市场、天长路等五条商业街和中环国际广场等八大商业综合体，加快推进华东农贸食品城、瑶海农机大市场、滁州国际商城、奥特莱斯直销广场、滁州国际车城、扬子农贸大市场建设，开工建设7家汽车4S店，争取红星美凯龙、沃尔玛、家乐福、钢材大市场等更多大型商贸物流企业进驻；县市区要进一步完善商业网点规划，发展特色物流市场，加强意大利名品商城、金太阳建材市场、天润城等县市区重点专业市场建设。积极发展第三方物流，建立公共物流信息平台，加快融入长三角物流圈。

　　激发金融服务业的活力。积极发展金融机构。努力引进股份制商业银行，支持徽商银行在县市区设立支行，积极引进战略投资者发展农村合作银行、村镇银行、小额贷款公司、贷款担保机构、典当机构和保险业，发展和利用资本市场，争取新组建农村合作银行和村镇银行各2至3家，每个县市区和市本级都要至少新发展小额贷款公司和贷款担保公司1家以上。积极争取市外驻滁金融机构扩大投放、地方性金融机构增资扩股，不断创新金融产品，改善金融服务，确保新增贷款超过全省平均水平。加强金融生态环境建设，保障金融业健康发展。

　　增强房地产业的动力。认真落实稳定房地产市场的政策措施，进一步调整住房供应结构，加快安置房、经济适用房和中低价位商品房开发建设，支持普通商品住房消费，鼓励居民购买自住房和改善性住房，积极发展旅游房地产、工业房地产和商业房地产，促进房地产业健康发展。今年要完成房地产投资80亿元，开工建设各类住房300万平方米，分别比上年增长21%和20%。

　　强化服务外包产业的推力。服务外包产业是新一轮国际产业转移的重点，是现代服务业的高端，是国内外发展低碳经济和新一轮产业布局的重头戏。就如同30年前发展加工贸易一样，谁能抢占先机，谁就抓住了未来对外贸易的制高点。要抓住稍纵即逝的机遇，力推服务外包发展。抓紧制定产业规划，依托市开发区规划建设滁州软件园和省级服务外包示范园区，积极支持康佳呼叫中心在滁州的发展，争取今年在引进服务外包企业上取得突破。

　　同时，还要加快发展科技和信息服务业，加强公共技术平台和信息基础设施建设，推进电子商务；加快发展商务服务业，鼓励发展法律、会计、审计、广告、咨询、评估等行业，积极引进总部经济；积极发展农业和农村服务业，加强农村金融、技术、信息服务，深入推进“万村千乡”市场工程和“新网工程”，大力发展农村连锁经营，活跃农村流通；探索发展居民服务业，开展养老托幼、清洁卫生、家庭教育等便民利民服务。狠抓园区、项目、企业、品牌四大服务业发展基础工程。加快建设滁州物流园、上海交大科技园、大琅琊山旅游度假区，规划建设城南中央商务区，编制一批重点服务业发展项目，扶持全市服务业30强，打造一批知名服务业品牌。今年起将服务业发展纳入政府目标考核范围。

　　（五）突出机制创新，在增强原动力上取得新突破

　　体制机制创新是发展的原动力。要按照中央和省统一部署推进体制改革，立足滁州实际开展机制创新，通过改革创新强化后发优势。

　　探索大部制的工作机制。在招商引资、园区建设、企业改革、旅游开发、市场建设、现代农业发展等领域全面推行大滁城建设指挥部的工作机制，实现兵团式作战和扁平化管理，全面推行“一线工作法”和挂图作战方式，加强工作调度，强化督查督办，打破部门分割，提高办事效率，确保各项工作加速推进。同时，正确处理好指挥部与部门职能之间的关系，把两大作用都充分发挥好。

　　完善大服务的政务平台。加强“两线”、“两中心”等政府公共服务平台建设，深化“两集中、两到位”改革，依法精简行政审批项目，优化审批流程，提高服务效率；巩固和完善企业登记注册、基建联合审批验收等四大平台，切实提高运行效率和服务质量；推行和完善帮办服务，创造良好的服务环境。

　　激发大发展的改革动力。深入开展改革攻坚年活动，强化重点领域改革攻坚。继续推进国有集体企业改革，启动47家国有企业改革工作，其中市直企业15家，确保全面完成省下达的“十一五”企业改革任务。大力开展投融资机制创新，分级整合各地城投、交投、国资公司等资源，做大政府融资平台，为经济发展提供更有力的资金支持。全面完成天长、琅琊的基层医药卫生体制改革试点工作，并积极稳妥地在全市推开，建立起政府举办的基层医疗卫生机构公益性管理体制，逐步取消“以药养医”。深入推进农村改革，继续深化政府预算管理体制改革、城市管理体制改革、行政审批体制改革等，实行事业单位绩效工资制度，抓好惠民直达工程试点，开展国有林场经营管理体制改革试点。

　　构建大开放的竞争格局。加快筹建滁州海关和商检局。认真落实促进外贸发展的政策，鼓励企业拓宽出口领域，优化出口产品结构和市场结构。加强利用外资工作，改变利用外资在全省相对落后的局面。加快发展非公有制经济，在大招商的同时，高度重视扶持本土企业发展壮大。制定《滁州市市区鼓励投资暂行办法》，加强各类创业园区建设，鼓励本地资本就地创业和扩大投资。

　　（六）突出民生改善，在增强和谐力上取得新突破

　　把握以人为本这个科学发展观的核心，在促进经济增长的同时，把改善民生放在更加突出的位置，不断发展社会事业，提高保障水平，让广大群众共享发展成果。

　　社会事业要有新进步。完善义务教育经费保障机制，不断加大教育投入，优化教育布局，改善农村地区和薄弱学校办学条件，促进教育均衡发展。实施中小学校校舍安全工程，完成60%以上的加固改造任务。着力推进职业教育大发展，加快建设科教园区，提高县市区职业学校办学水平。积极支持安徽科技学院、滁州学院、滁州职业技术学院建设，加强中等专业学校的整合和大专体制的申报工作。加快市第一医院、中西医结合医院医技大楼和二院新院区等重点工程建设，提高医疗水平。加强社区卫生服务机构和乡镇卫生院、村卫生室标准化建设，提高硬件水平。加强公共卫生管理，深入开展爱国卫生运动，认真做好重大疫病防控工作。加强公共文化场所和文化设施建设，扎实开展群众性文化体育活动和精神文明创建活动。加强文物和非物质文化遗产保护和开发利用工作。加快数字电视转换工作，办好广播电视事业。做好第六次人口普查工作。加强国防后备力量建设，深入开展国防教育，做好优抚安置，重视人防工作，争创全国双拥模范城“四连冠”。同时，抓好统计、气象、地震、外事、侨务、对台、档案、保密、民族宗教、地方志、关心下一代等工作，支持工会、共青团、妇联、工商联、科协、红十字会工作，发展妇女儿童、老龄人、残疾人等各项事业。

　　服务管理水平要有新提高。推进无线宽带城市建设，提高城市信息化和数字化管理水平。加强社会治安综合治理，严厉打击各类违法犯罪和传销等活动。认真做好信访工作，强化维稳和信访责任制，加强重大事项社会稳定风险评估，认真排查调处矛盾纠纷，积极化解不稳定因素，完善应急管理工作机制，提高预防和处置突发事件能力。加强基层民主法治建设，深入开展“法治在滁州”创建活动，全面完成“五五”普法任务。加强社区建设，强化流动人口管理服务。严格落实安全生产责任制和安全隐患挂牌督办制，深入整治交通、建筑、消防、危化、矿山、卫生等领域的突出隐患和问题。加强物价和市场监管，整顿和规范市场秩序。

　　就业再就业要有新拓展。认真落实促进就业的各项政策，稳定企业用工。加强就业指导服务、城乡劳动力就业培训和人力资源市场建设，做好城镇零就业家庭帮扶工作，稳步扩大政府提供的公益性就业岗位。认真调处劳动争议，构建和谐劳动关系。今年争取新增就业达到3.3万人。

　　民生福祉要有新改善。在继续抓好去年省市民生工程（招录医务人员除外）的同时，建设一批县级儿童福利中心、留守流动儿童活动室、留守儿童之家、标准化光荣院，在每个乡镇建设生活垃圾收集和处理设施，不折不扣地完成省市36项民生工程实施任务。同时按照尽力而为、量力而行的原则，加大民生投入。要突出抓好社会保障工作，进一步完善农村新型合作医疗、城镇居民基本医疗保险、城乡低收入家庭社会保障、失地农民社会保障等各项社会保障制度，稳步扩大职工医疗、养老、失业、工伤、生育保险覆盖面，提高社会保障水平。

　　（七）突出人资环统筹，在增强持续力上取得新突破

　　统筹人口、资源和环境保护是滁州科学发展、可持续发展的必然选择，是转变滁州发展方式的根本要求。加强人口和计划生育工作，强化性别比治理，狠抓基层基础工作，突出抓好后进乡村治理，建立健全长效机制，确保基层线、党政线、部门线和流动人口计划生育工作取得新成效，确保年终考核在全省位次前移。按照“整合矿产资源、提高开采标准”的思路，推进矿产资源高水平开发利用。加强土地集约节约利用，依法处置闲置土地和违法用地。以创建国家级生态环保城市为目标，切实加强环境保护，严格执行项目建设“三同时”制度，坚决把污染项目拒之门外。做好节能减排工作，鼓励企业采用节能降耗技术，大力发展循环经济，提高资源利用效率。提高排污企业在线监控水平，加强重点污染企业监管，确保被省挂牌企业全面摘牌。加强城市污水处理管网建设，启动市污水处理厂二期建设，积极推广雨污分流。在电力、建材、纺织、化工等重点能耗产业和企业，深入开展节能专项行动，积极推广节煤、节电、节水等新技术和节能新材料，加快淘汰落后产能。抓好凤阳西部建材群、石英砂加工整治工作。积极探索农村集镇垃圾处理办法，加强秸秆还田和综合利用，积极推广农村清洁能源，加强森林和水资源保护，强化饮用水源地管理，整治农村面源污染。

　　三、建设奋发有为 人民满意的政府

　　滁州正面临历史上难得的突破机遇，也面临着更加激烈的竞争环境。政府承担着带领全市人民加快发展的重大责任，要敢于担当，把为人民谋利益作为最高任务，把对人民负责作为最高准则，把让人民满意作为最高追求，不断加强自身建设，保持好奋发有为的精神状态，在崛起跨越的进程中永不停步。

　　建设学习政府，提高创新力。突破式发展需要突破型能力。要推进学习型机关建设，加强公务员队伍学习培训，帮助政府系统全体人员牢固树立科学发展新理念，拓展国际视野新思维，把握外部发展新变化，提升解放思想新境界，顺应人民群众新期待，不断提高抢抓机遇的能力、创新发展的能力、高效落实的能力和破解难题的能力，善于把上级部署与滁州实际更好地结合，把满腔热情与务实态度更好地结合，把各项决策与群众利益更好地结合，开创发展的新局面。

　　建设法治政府，提高公信力。深入开展领导干部学法活动，增强各级政府机关工作人员依法行政意识。坚持依法办事，严格按照法定程序和权限履行职责、管理经济和社会事务。加强行政执法监督，做好行政复议工作，规范行政行为。完成市县政府机构改革，调整政府职能，加快培育中介服务组织，把政府的主要精力转移到社会管理、公共服务和创造良好发展环境上来。切实落实招商引资中的承诺，取信于商；切实兑现在土地征用、企业改革、城市拆迁工作中的承诺，取信于民，做到言必行、行必果。自觉接受人大及其常委会的法律监督和工作监督，主动接受政协的民主监督，办好人大议案、代表建议、人大常委会审议意见和政协提案，认真听取各民主党派、工商联、人民团体意见。

　　建设为民政府，提高亲和力。坚持以人为本，在决策和执行中充分关注群众的根本利益，多讲群众听得懂的“地方话”，多做为群众排忧解难的简化式。坚持问计于民、问政于民、问需于民，对涉及民生的重要事项及时组织听证，强化政务公开，保障人民群众的知情权、参与权和监督权。建立联系基层制度，确保经常到基层，进村入户、进园入企，广泛听民意、知民情、解民忧。办好市长热线，开展上网交流，畅通与群众和网民的沟通渠道。不断改善对企业的服务，坚决打击吃拿卡要企业的行为，依法保护企业的合法权益。

　　建设服务政府，提高执行力。加强公务员队伍管理，切实增强服务基层、服务群众、服务企业的公仆意识。深入开展机关效能提升年活动，加强机关作风建设，治庸提能、治懒增效、治散正气，大力整治机关中存在的懒、散、慢、庸、拖等不良风气和不作为、乱作为、慢作为现象，努力营造人心思进的生动局面。完善目标绩效考评体系，健全激励和约束机制，实名考核、实名问效、实名问责、实名奖惩。切实精简会议、压缩文件、减少事务性活动，把更多的时间和精力放在抓大事、解难事、办实事上。

　　建设清廉政府，提高凝聚力。从严治政、标本兼治、惩防并举，全面加强政府系统惩治和预防腐败体系建设，全面落实政府系统党风廉政责任制。坚持用制度管人管钱管事，加强制度创新，进一步完善招投标管理体制，强化对重大工程、重点领域、重要环节的监管，加强行政监察和审计监督，从源头上防止腐败。坚决纠正部门和行业不正之风，严肃查处各级政府工作人员违法违纪行为。大力发扬艰苦朴素的作风，厉行勤俭节约，坚决反对讲排场、比阔气的行为，切实把资金用在最需要的地方。

　　今年是“十一五”规划最后一年，在抓好以上工作的同时，要着眼于滁州未来5至10年跨越发展、科学发展、和谐发展，加强对市情的深度研究，加强对重大问题的前瞻性研究，找准国家政策和本地实际的结合点，找准滁州跨越式发展的突破点，找准支撑滁州经济增长的爆发点，集思广益，精心编制“十二五”规划，同时积极争取将我市的重大战略谋划与战略性新兴项目纳入省级以上总体规划或专项规划之中。

　　各位代表，新的使命崇高而伟大，新的任务光荣而艰巨。时代在召唤我们，人民在期待我们。让我们紧密地团结在以胡锦涛同志为总书记的党中央周围，高举中国特色社会主义伟大旗帜，全面贯彻落实科学发展观，在省委省政府和市委的坚强领导下，解放思想，奋力拼搏，不畏艰险，不惧挑战，不辱使命，为推动滁州经济社会又好又快发展，为早日实现“千亿滁州”而努力奋斗！

　　谢谢大家！
